# Tissue Factor-Expressing Tumor-Derived Extracellular Vesicles Activate Quiescent Endothelial Cells *via* Protease-Activated Receptor-1

**DOI:** 10.3389/fonc.2017.00261

**Published:** 2017-11-02

**Authors:** Sara P. Y. Che, Jeannie Y. Park, Tracy Stokol

**Affiliations:** ^1^Meinig School of Biomedical Engineering, Cornell University, Ithaca, NY, United States; ^2^Department of Population Medicine and Diagnostic Sciences, College of Veterinary Medicine, Cornell University, Ithaca, NY, United States

**Keywords:** cancer, cell-derived microparticles, exosomes, protease-activated receptors, thromboplastin

## Abstract

Tissue factor (TF)-expressing tumor-derived extracellular vesicles (EVs) can promote metastasis and pre-metastatic niche formation, but the mechanisms by which this occurs remain largely unknown. We hypothesized that generation of activated factor X (FXa) by TF expressed on tumor-derived EV could activate protease-activated receptors (PARs) on non-activated endothelial cells to induce a pro-adhesive and pro-inflammatory phenotype. We obtained EV from TF-expressing breast (MDA-MB-231) and pancreatic (BxPC3 and Capan-1) tumor cell lines. We measured expression of E-selectin and secretion of interleukin-8 (IL-8) in human umbilical vein endothelial cells after exposure to EV and various immunologic and chemical inhibitors of TF, FXa, PAR-1, and PAR-2. After 6 h of exposure to tumor-derived EV (pretreated with factor VIIa and FX) *in vitro*, endothelial cells upregulated E-selectin expression and secreted IL-8. These changes were decreased with an anti-TF antibody, FXa inhibitors (FPRCK and EGRCK), and PAR-1 antagonist (E5555), demonstrating that FXa generated by TF-expressing tumor-derived EV was signaling through endothelial PAR-1. Due to weak constitutive PAR-2 expression, these endothelial responses were not induced by a PAR-2 agonist peptide (SLIGKV) and were not inhibited by a PAR-2 antagonist (FSLLRY) after exposure to tumor-derived EV. In conclusion, we found that TF-expressing cancer-derived EVs activate quiescent endothelial cells, upregulating E-selectin and inducing IL-8 secretion through generation of FXa and cleavage of PAR-1. Conversion of resting endothelial cells to an activated phenotype by TF-expressing cancer-derived EV could promote cancer metastases.

## Introduction

The majority of epithelial tumors upregulate tissue factor (TF) (1) and tumor-derived extracellular vesicles (EVs), including exosomes (30–150 nm) and membrane-derived microparticles (150–1,000 nm), expressing TF antigen and procoagulant activity have been isolated from plasma of cancer patients (2–4) and conditioned media from cell lines (5–7). The pathologic relevance of tumor-derived EV is an active area of investigation, specifically in cancer-associated thrombosis (5) and cancer progression (8). In one study, TF-expressing tumor-derived EV promoted pre-metastatic niche formation and metastasis in mice by initiating coagulation and recruiting bone marrow-derived monocytic cells (8), which was prevented by inhibition of TF-triggered thrombin generation.

Normal quiescent endothelial cells act as a barrier to cancer metastasis, but pro-inflammatory mediators can activate endothelial cells to induce expression of pro-inflammatory adhesion molecules and secretion of pro-inflammatory cytokines. Such endothelial responses can potentially promote cancer metastasis by facilitating recruitment of tumor cells or bone marrow-derived hematopoietic cells involved in pre-metastatic niche formation (8–10). Activation of endothelial cells by EV has been observed previously, resulting in increased vascular permeability, angiogenesis, and cancer metastasis (10–14); however, the mechanisms remain largely unknown.

Tissue factor–factor VIIa (FVIIa)–factor Xa complexes on the surface of tumor-derived EV can activate endothelial cells by cleaving protease-activated receptors (PARs). Endothelial cells express all four PAR, although PAR-4 expression in human umbilical vein endothelial cells (HUVEC) varies between studies (15, 16). Thrombin, which is generated by TF-expressing EV in plasma, preferentially cleaves PAR-1 (17, 18), whereas the TF–FVIIa–activated factor X (FXa) complex cleaves PAR-1 and PAR-2 (19, 20) at physiological concentrations (21). PAR-1 is highly expressed by quiescent endothelial cells, whereas PAR-2 is upregulated in these cells only after stimulation with inflammatory mediators or exposure to hypoxic conditions (22, 23). Purified FXa has been shown to activate endothelial cells *in vitro*, inducing the secretion of pro-inflammatory cytokines and upregulating adhesion molecules (24, 25). This effect could be mediated by both PAR-1 and PAR-2; however, FXa is presumed to signal preferentially through PAR-2 (21, 22, 26, 27).

In this study, we hypothesized that TF-expressing tumor-derived EV would activate non-activated endothelial cells, inducing upregulation of adhesion molecules and secretion of cytokines in a PAR-1 and PAR-2 dependent manner. We found that TF-expressing tumor-derived EV from breast and pancreatic cancer cell lines activated endothelial cells within 6 h of exposure, and this activation required TF-mediated generation of FXa. The endothelial responses were mediated through PAR-1 but not PAR-2. Our results indicate that PAR-1 is the main receptor for eliciting TF-expressing tumor-derived EV responses in unstimulated endothelial cells.

## Materials and Methods

### Cell Lines and Cell Culture

Breast cancer cell line—MDA-MB-231 (ATCC NCI-PBCF-HTB26, Manassas, VA, USA)—and pancreatic cancer cell lines—BxPC3 (ATCC CRL-1687) and Capan-1 (ATCC HTB-79)—were cultured in Dulbecco’s Modified Eagle Medium, Roswell Park Memorial Institute-1640 and Iscove’s Modified Dulbecco’s Medium (Corning, Corning, NY, USA), respectively. Media were completed with 10% (v/v) fetal bovine serum (20% for Capan-1; Atlanta Biologicals, Norcross, GA, USA) and 100 U/ml penicillin–streptomycin (Invitrogen, Carlsbad, CA, USA). E4+ HUVEC (gift from Dr. Seandel, Cornell-Weill Medical) were cultured in EGM-2 (Lonza, Basel, Switzerland). Cells were maintained at 37°C and 5% CO_2_.

### EVs from Tumor Cell Lines

At 70–90% confluency, tumor cells were serum starved overnight. Tumor-conditioned media were centrifuged at 300 × *g* for 5 min and 2,500 × *g* for 20 min to remove cells and debris, then 100,000 × *g* for 70 min at 4°C in an Optima L-90K ultracentrifuge (Beckman Coulter, Pasadena, CA, USA) to pellet EV. The pellet was resuspended in phosphate-buffered saline (PBS) and used immediately.

### Nanoparticle Tracking Analysis of EVs

Samples were diluted with PBS and analyzed using in a Malvern NanoSight NS300 (Westborough, MA, USA) with 3 × 60 s runs at ambient temperature. EV size distribution and concentration were determined by manufacturer’s software (V3.0, camera level at 16 and threshold at 10).

### Atomic Force Microscopy of EVs

Extracellular vesicles, prepared as above, were resuspended in water and then centrifuged at 100,000 × *g* for 2 h at 4°C. The pellet was then resuspended in water. Atomic force microscopy was conducted at the University of Texas Houston AFM Core Facility. A 10 μl sample of EV suspension (at a concentration of 2.5 × 10^11^ EV/ml as determined by nanoparticle tracking analysis) was incubated for 10 min on freshly cleaved mica surfaces (V1 mica disks, Ted Pella, Redding, CA, USA). Excess liquid was then removed, and the sample was allowed to dry at room temperature. All samples were freshly prepared and imaged immediately. Atomic force microscopy was performed using RTESP cantilevers (*f*_o_ = 237–289 kHz, *k* = 20–80 N/m, Bruker, Santa Barbara, CA, USA) and BioScope II AFM (Bruker). Images (5 μm × 5 μm) were taken using tapping mode operated in air at a scan rate of 0.6 Hz, then analyzed using NanoScope Analysis (V1.40, Bruker).

### Procoagulant Activity of EVs

Tumor-derived EVs were diluted 1:5 in HEPES buffer (10 mM HEPES, 137 mM sodium chloride, 5 mM calcium chloride, 4 mM potassium chloride, 10 mM glucose, 0.5% bovine serum albumin, and pH 7.4) and incubated with 1 nM FVIIa and 75 nM FX for 15 min at 37°C. The concentration of FX does not limit the reaction because the highest concentration of generated FXa was below 20 nM in our study. A chromogenic substrate (40 μM Spectrozyme, Sekisui Diagnostics, Lexington, MA, USA) was added, and color change was measured at 405 nm every 15 s for 10 min. Factor Xa generation was calculated based on a standard curve of purified FXa. To account for TF-independent FX activation, control values (with FX only) were deducted from reported values.

### Exposure of Endothelial Cells to EV

Endothelial cells were washed and put into serum-free media for 1 h. To determine the role of TF-mediated signaling, EVs were pretreated with FVIIa and FX (10 and 150 nM, respectively, unless otherwise noted; Hematologic Technologies, Essex Junction, VT, USA). To inhibit TF, EVs were pretreated with 10 μg/ml HTF-1 (BD Bioscience, Franklin Lake, NJ, USA) or isotype control for 30 min. As positive controls, endothelial cells were treated with bovine thrombin (1 U/ml, Sigma-Aldrich, St. Louis, MO, USA), or FXa (80 nM, Hematologic Technologies). To inhibit FXa, FPRCK or EGRCK (40 μM, Hematologic Technologies) was added. To determine the role of endothelial PAR-1 and PAR-2 signaling, PAR-1 or PAR-2 agonist peptides (100 μM; TFLLR, Sigma-Aldrich, and SLIGKV, Abcam, Cambridge, UK, respectively) and scramble peptides (RLLFT, American Peptide Company, Sunnyvale, CA, USA, and VKGILS, Bachem, Torrance, CA, USA, respectively) were used with amastatin (10 μM, Santa Cruz Biotechnology, Dallas, TX, USA) to prevent peptide degradation (28). To inhibit PAR-1 and PAR-2, endothelial cells were pretreated with 0.1 μM E5555 (Axon Med Chem, Reston, VA, USA) and 50 μM FSLLRY (Bachem) for 30 min at 37°C. We did not use SCH79797, which induced cell death at the concentration required for PAR-1 inhibition (29, 30). Untreated endothelial cells were used as negative controls.

### Cell-Based ELISA for Adhesion Molecule Expression

Endothelial cells were exposed to EV or agonists for 6 h at 37°C, then fixed with 2% paraformaldehyde for 15 min. Wells were blocked with 5% normal goat serum (Jackson ImmunoResearch, West Grove, PA, USA) for 30 min, then incubated with 1 μg/ml antibody against E-selectin (1.2B6, Abcam) overnight at 4°C. Wells were incubated with horseradish peroxidase-conjugated secondary antibody (1:150, Thermo Scientific, Waltham, MA, USA) for 1 h, then with 3,3′,5,5′-tetramethylbenzidine substrate (Thermo Scientific) for 30 min. Washing with PBS was performed between incubation steps. Sulfuric acid (2 N) was added, and absorbance was read at 450 nm (Molecular Devices SpectroMax M3, Sunnyvale, CA, USA). Data were normalized to untreated endothelial cells due to baseline fluctuations and presented as fold change.

### ELISA for Cytokine Secretion

Cultured media from endothelial cells was collected and stored at −80°C. Samples were centrifuged at 15,000 × *g* for 10 min at 4°C to remove debris and aggregates. The interleukin-8 (IL-8) concentration (linear range 31.2–2,000 pg/ml) was measured using commercial kits according to the manufacturer’s instructions (R&D Systems ELISA DuoSets, Minneapolis, MN, USA) and was not normalized to untreated endothelial cells.

### Flow Cytometry for Cell Surface TF and PAR Expression

Cells (5 × 10^5^) were suspended in PBS with 1% bovine serum albumin and incubated with primary antibodies (20 μg/ml anti-TF antibody: TF9-5B7 hybridoma from Dr. James Morrissey, University of Illinois; 4 μg/ml anti-PAR-1 antibody: ATAP2, Santa Cruz; 4 μg/ml anti-PAR-2 antibody: SAM11, Santa Cruz) or isotypes for 30 min on ice. MDA-MB-231 was used as a positive control for PAR-1 and PAR-2 labeling. Alexa-488-conjugated secondary antibody (10 μg/ml, Invitrogen) was added to the cells for 30 min on ice. Cells were resuspended in PBS for flow cytometry (FACSCalibur, Becton Dickenson, Franklin Lakes, NJ, USA). Data (10,000 events) were analyzed (v10 FlowJo, FlowJo, Ashland, OR, USA).

### Immunofluorescent Microscopy for Endothelial E-Selectin and PAR Expression

Endothelial cells were grown on fibronectin-coated coverslips, then exposed to EV and fixed as above. Cells were stained with an antibody against E-selectin, PAR-1, or PAR-2 (10 μg/ml) for 30 min, followed by an Alexa-488-conjugated secondary antibody for 30 min. Coverslips were sealed using mounting medium with DAPI (VECTASHIELD, Vector Laboratories, Burling Game, CA, USA) and imaged (Nikon Eclipse TE2000-U, Melville, NY, USA; Photometrics CoolSNAP HQ_2_ camera, Tucson, AZ, USA).

### Immunoblot Analysis for TF Antigen, CD9 and Flotillin Expression, and ERK Phosphorylation

Cells and EV pellet were lysed, then stored at −80°C. Lysates (30 μg) were resolved using 10% SDS/PAGE, and proteins were transferred to nitrocellulose membrane. Membranes were incubated with primary antibody (TF: EPR8986, Abcam; phospho-ERK: E10, actin: 8H10D10, and flotillin: C42A3, Cell Signaling Technologies, Danvers, MA, USA; ERK: 16/ERK, BD Biosciences; CD9: C-4, Santa Cruz, Santa Cruz, CA, USA) overnight at 4°C, and then horseradish peroxidase-conjugated secondary antibody for 1 h. Signal was detected using chemiluminescent substrate (Thermo Scientific) and imaged by film or imager (BioSpectrum Imaging System, UVP, Upland, CA, USA). Experiments were duplicated two to three times using different lysates and EV preparations.

### Cell Viability Assay

Endothelial viability was determined using a commercial kit based on ATP quantification (CellTitre Glo, Promega, Madison, WI, USA). Data were normalized to untreated endothelial cells and presented as a percentage.

### Statistical Analysis

Data were parametric and represented as mean ± SD. Means of different treatments were compared with a Student’s *t*-test and α = 0.05 using Prism 6.0 (GraphPad, La Jolla, CA, USA).

### Ethics Approval Statement

No ethics approval statement was required for the study.

## Results

### Tumor-Derived EVs Express TF Antigen and Procoagulant Activity

**Figure 1:**
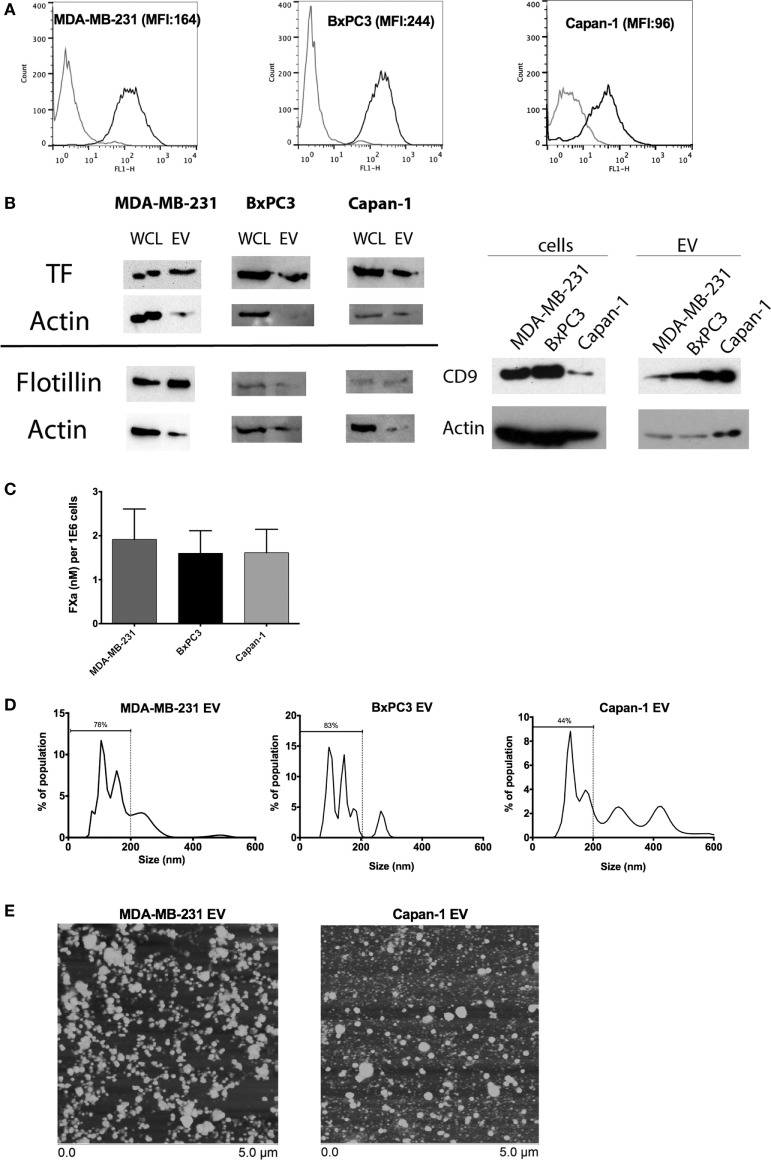
Characterization of tissue factor (TF) antigen and procoagulant activity on tumor cell lines and secreted extracellular vesicle (EV). **(A)** Representative histograms and mean fluorescence intensity (MFI) of surface TF antigen expression on MDA-MB-231, BxPC3, and Capan-1 as determined by flow cytometry with an antibody against TF (black line) and an isotype control (gray line). **(B)** TF antigen expression was determined in whole cell lysates (WCL) and EV using immunoblotting. Flotillin and CD9 were used to verify the presence of EV on separate immunoblots. Actin was used as a loading control. **(C)** Procoagulant activity of tumor-derived EV (normalized per million of original tumor cells) was measured using an amidolytic assay for activated factor X (FXa) activation. **(D)** Representative size distribution of tumor-derived EV as characterized by nanoparticle tracking analysis (NTA). All experiments were done in triplicate. **(E)** Atomic force microscopy showed that EV preparations from MDA-MB-231 and Capan-1 consisted of a mixture of small and large vesicles, compatible with exosomes and microvesicles. The preparations from Capan-1 contained more larger vesicles, consistent with the NTA results.

Tissue factor antigen was expressed on the surface of MDA-MB-231, BxPC3, and Capan-1 cells, as shown by flow cytometric analysis (Figure 1A). Similarly, EV derived from these tumor cell lines expressed TF antigen on immunoblotting, with flotillin and CD9 expression confirming successful isolation of EV (Figure 1B; Figure S1 in Supplementary Material). EV from all three cell lines generated similar amounts of FXa (Figure 1C). The size distribution of EV derived from MDA-MB-231 and BxPC3 cells was similar, consisting mostly of small vesicles (approximately 80% of population less than 200 nm), with a small amount of large vesicles, likely membrane-derived microvesicles (>200 nm) (Figure 1D). However, Capan-1 cells generated more large microvesicles (close to 50%). The size distribution was verified using atomic force microscopy (Figure 1E), which showed vesicles with a range of size (~50–300 nm). We observed more large individual vesicles from Capan-1 cells, supporting the nanoparticle tracking analysis. The size of released EV has been shown to vary among different cell lines derived from different tissues and is dependent on genes involved in vesicle trafficking (31).

### Endothelial Cells Exhibit High Constitutive Expression of PAR-1 and Weak Expression of PAR-2

**Figure 2:**
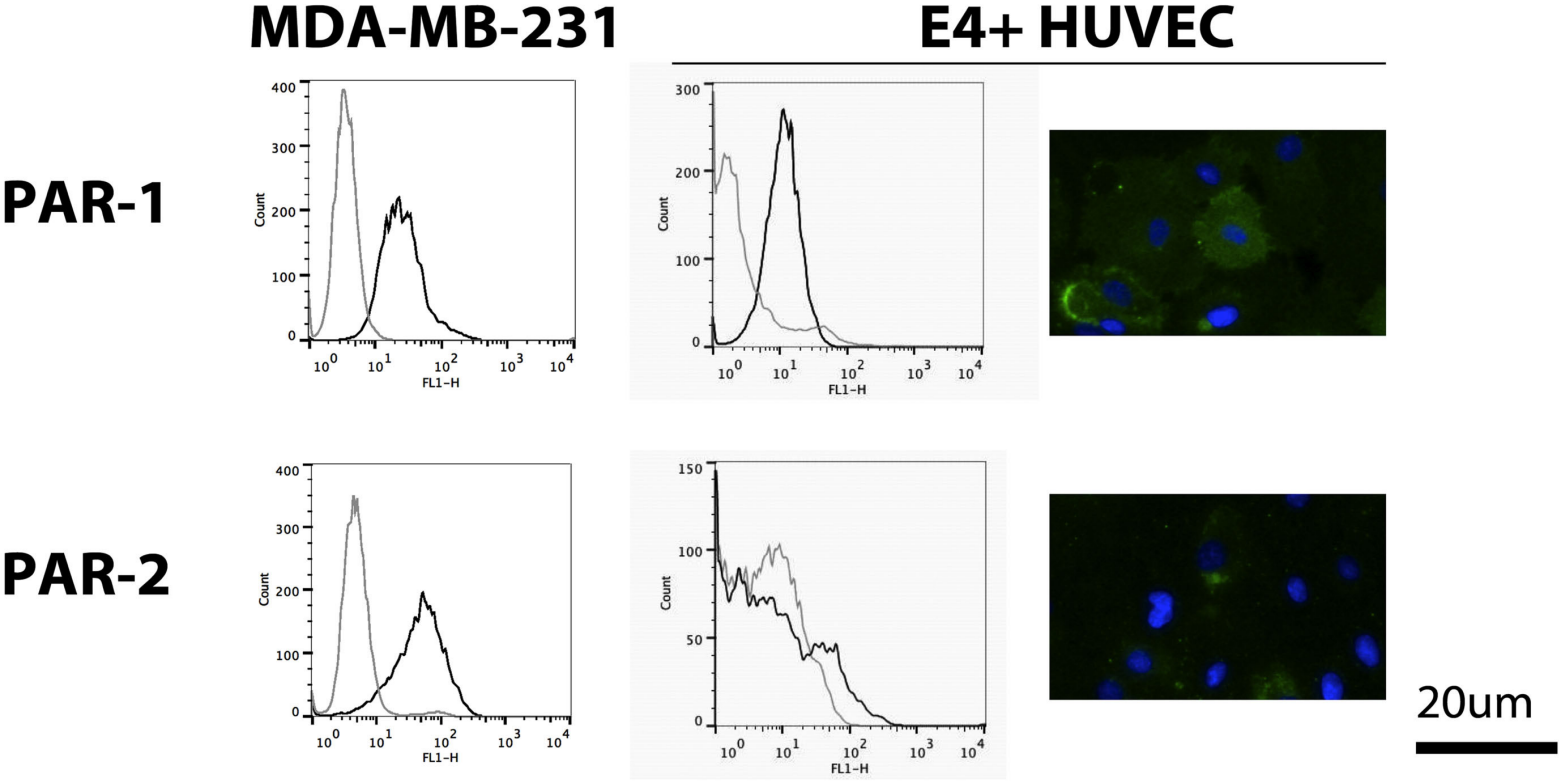
Protease-activated receptor (PAR) expression on E4+ human umbilical vein endothelial cells (HUVEC). Expression of PAR-1 and PAR-2 on E4+ HUVEC, using MDA-MB-231 breast cancer cells as a positive control, was evaluated using flow cytometry (black line: protein of interest; gray line: isotype control) and immunofluorescent microscopy (green: protein of interest; blue: nuclear counterstain).

We evaluated PAR-1 and PAR-2 expression on non-activated E4+ HUVEC with flow cytometry and immunofluorescent microscopy. We found PAR-1 was highly expressed, whereas weak PAR-2 expression was only seen in a small proportion of E4+ HUVEC, as shown by the small peak to the right of the isotype control on flow cytometric analysis and few positive staining cells on immunofluorescent microscopy (Figure 2). To verify this phenotype was not a consequence of the ORF4 gene, we also tested HUVEC and found a similar pattern of expression for both PAR-1 and PAR-2 (Figure S2A in Supplementary Material), as reported previously (23). Strong surface expression of PAR-1 and PAR-2 was observed on MDA-MB-231 breast cancer cells with flow cytometry (Figure 2), which were used as a positive control (32).

### TF-Expressing Tumor-Derived EV Upregulate Endothelial E-Selectin Expression and Induce IL-8 Secretion in a TF-, FXa-, and PAR-1-Dependent, but PAR-2-Independent, Manner

**Figure 3:**
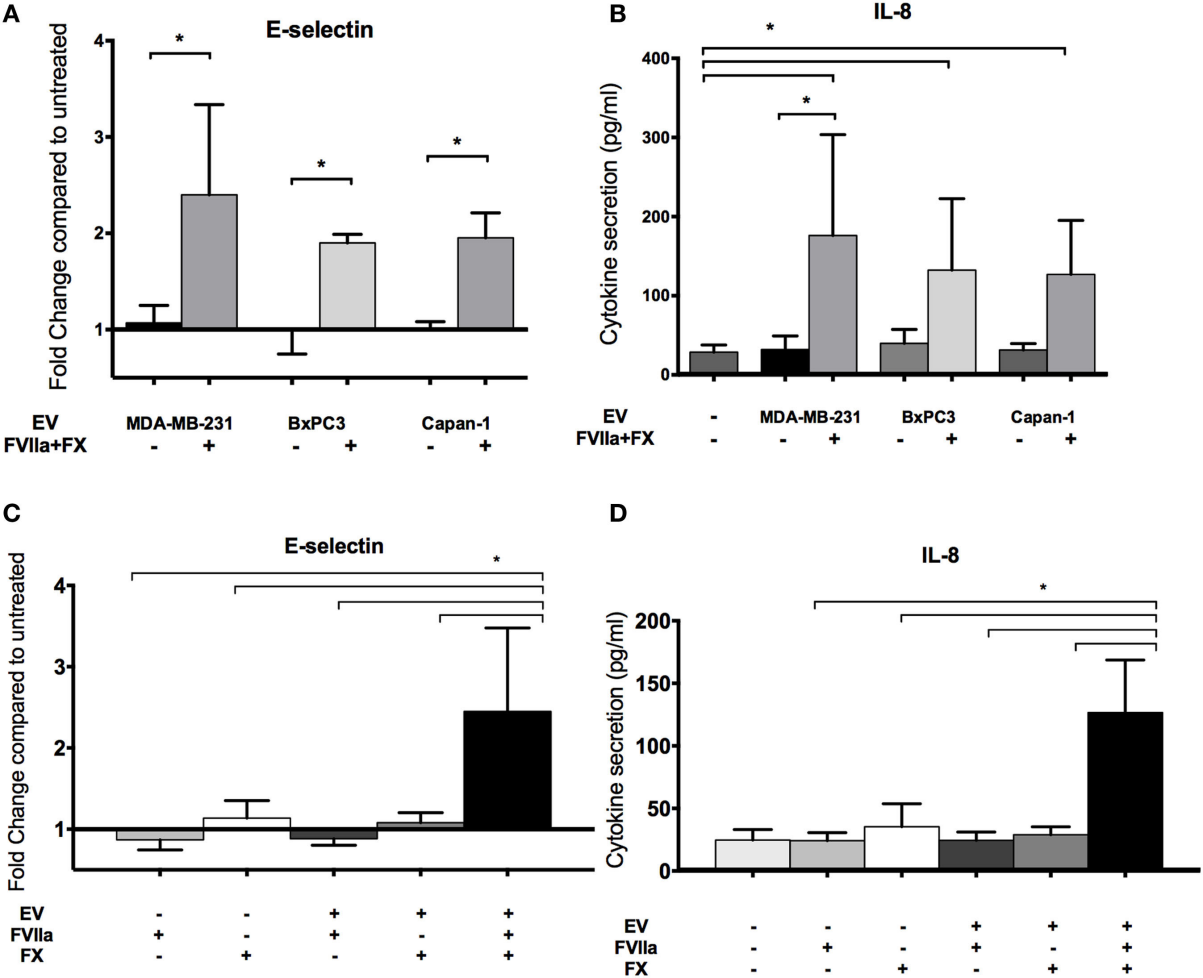
Increased endothelial E-selectin expression and interleukin-8 (IL-8) secretion after exposure to tumor-derived extracellular vesicle (EV) require factor VIIa (FVIIa) and FX. E4+ human umbilical vein endothelial cells were exposed to EV (with or without FVIIa and FX) derived from MDA-MB-231, BxPC3, and Capan-1 tumor cells for 6 h. **(A,C)** Endothelial E-selectin expression and **(B,D)** IL-8 secretion were evaluated using a cell-based ELISA and ELISA on conditioned media, respectively. Increased endothelial E-selectin expression **(A)** and IL-8 secretion **(B)** were observed when EVs were pretreated with 10 nM FVIIa and 150 nM FX. Both FVIIa and FX were required for these endothelial responses **(C,D)**. All experiments were repeated three to four times. *p < 0.05.

Endothelial cells were treated with EV derived from MDA-MB-231, BxPC3, and Capan-1 cell lines for 6 h. We observed an increase in endothelial E-selectin surface expression and IL-8 secretion only when EVs were pretreated with FVIIa and FX (Figures 3A,B). We saw similar upregulation in non-activated HUVEC with tumor-derived EV pretreated with FVIIa and FX (Figures S2B,C in Supplementary Material), indicating that these responses were not due to E4+ ORF gene insertion. Subsequent experiments were performed with MDA-MB-231-derived EV as our model tumor EV.

The EV-induced changes in endothelial cells required both FVIIa and FX, suggesting that generation of FXa by TF-FVIIa on EV is required (Figures 3C,D). In support of this, we found that FXa alone induced a mild increase in E-selectin expression and IL-8 secretion, although the response was far weaker than that induced by EV pretreated with FVIIa and FX (Figure S3A in Supplementary Material). This suggests that FXa generated on the surface of cell membranes is more effective than free FXa for inducing pro-inflammatory responses in endothelial cells.

**Figure 4:**
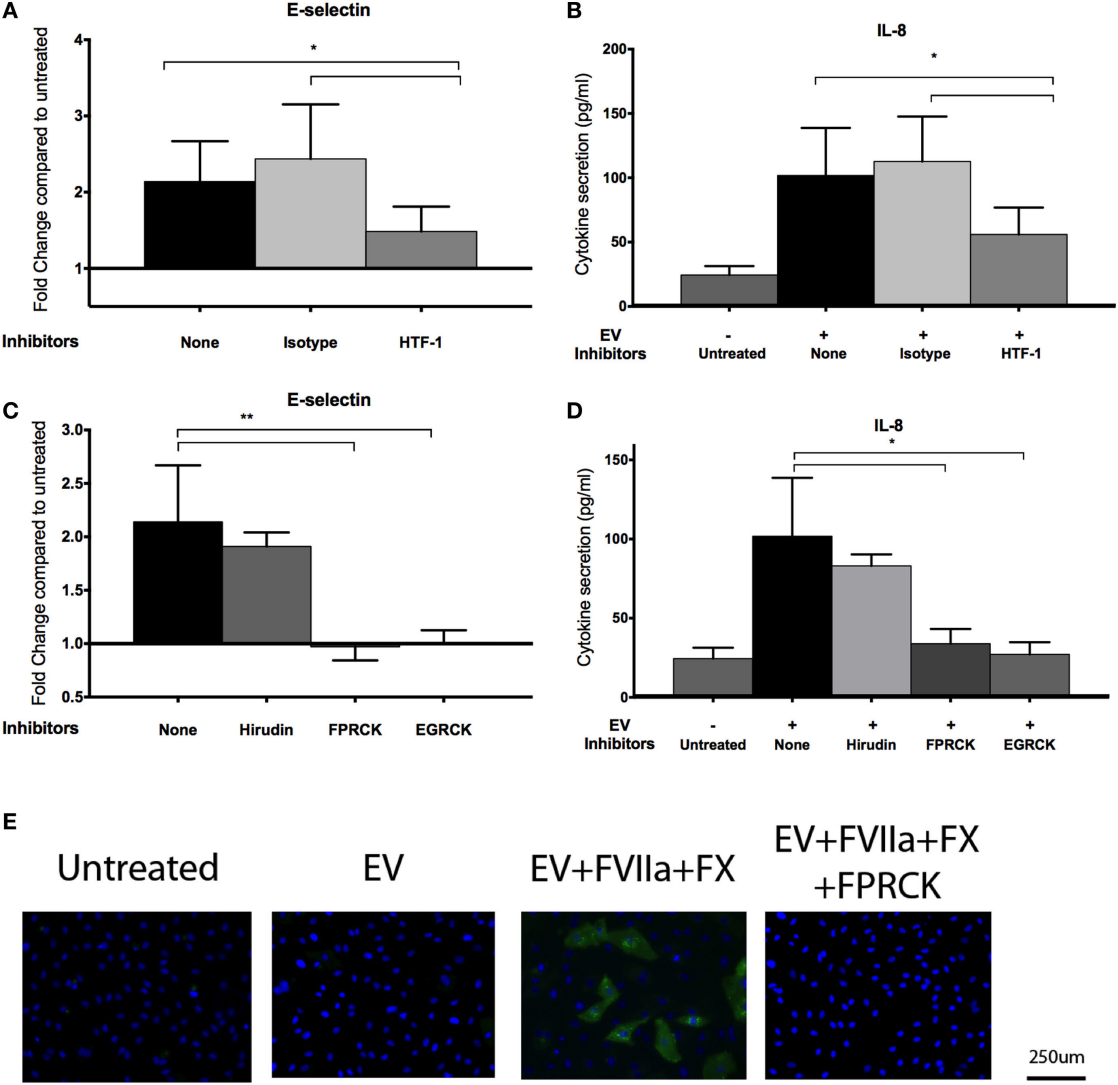
MDA-MB-231-derived extracellular vesicle (EV)-induced upregulation of endothelial E-selection expression, and interleukin-8 (IL-8) secretion is tissue factor (TF) and activated factor X (FXa) dependent. **(A,B)** To inhibit TF, MDA-MB-231-derived EVs were pretreated with 10 μg/ml HTF-1 (anti-TF antibody) or isotype control for 30 min before pretreatment with factor VIIa (FVIIa) and FX, followed by incubation with E4+ human umbilical vein endothelial cells (HUVEC) for 6 h with FVIIa and FX. E-selectin expression **(A)** and IL-8 secretion **(B)** were measured after the treatments (*n* = 6). **(C,D)** A thrombin inhibitor, hirudin (10 U/ml), and FXa inhibitors, FPRCK and EGRCK (40 μM), were added with the EV. E-selectin expression **(C)** and IL-8 secretion **(D)** were measured after the treatments (*n* = 3). *p < 0.05, **p < 0.01. **(E)** After incubation with MDA-MB-231-derived EV, E4+ HUVEC were fixed and immunostained with an antibody against E-selectin (green) to evaluate upregulation with immunofluorescent microscopy. Nuclei were counterstained blue with DAPI. Experiments were done in triplicate.

An antibody against TF (HTF-1) and FXa inhibitors (FPRCK and EGRCK) reduced and abolished upregulation of E-selectin and secretion of IL-8 in E4+ HUVEC, respectively (Figures 4A–D), supporting that these responses were mediated by TF-mediated generation of FXa. The FXa inhibitor, FPRCK and EGRCK, also inhibited endothelial responses elicited by purified FXa (Figures S3A,B in Supplementary Material). With immunofluorescent microscopy, E-selectin expression was observed on a subpopulation of E4+ HUVEC exposed to tumor-derived EV and was abolished with FPRCK (Figure 4E). Although we performed our experiments in serum-free media, traces of thrombin may be present. Thrombin has been shown to induce E-selectin expression and IL-8 secretion *via* cleavage of PAR-1 (33). Inclusion of 10 U/ml hirudin, a specific thrombin antagonist, abolished endothelial responses to thrombin (Figure S3C in Supplementary Material), but did not significantly reduce the responses to MDA-MB-231-derived EV pretreated with FVIIa and FX (Figures 4C,D).

**Figure 5:**
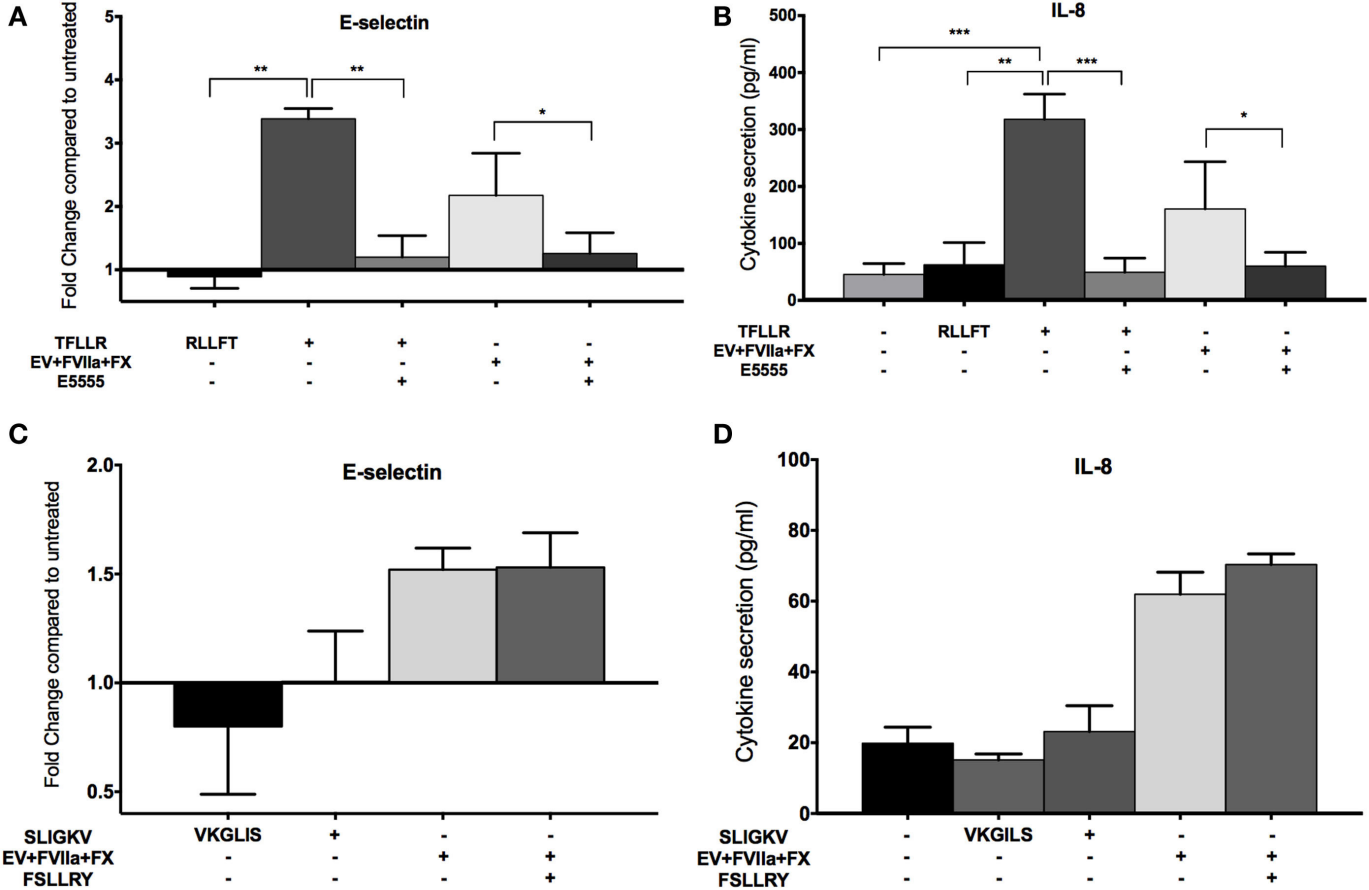
MDA-MB-231-derived extracellular vesicle (EV)-induced upregulation of endothelial E-selection expression, and interleukin-8 (IL-8) secretion is protease-activated receptor (PAR)-1 dependent, but not PAR-2 dependent. **(A,B)** E4+ human umbilical vein endothelial cells (HUVEC) were treated with 100 μM TFLLR (PAR-1 agonist with 100 μM scrambled peptide RLLFT as control) and MDA-MB-231-derived EV (pretreated with FVIIa and FX). To inhibit PAR-1, endothelial cells were pretreated with 0.1 μM E5555 for 30 min before exposure to EV. E-selectin expression **(A)** and IL-8 secretion **(B)** were measured after the treatments (*n* = 5). **(C,D)** E4+ HUVEC were treated with 100 μM SLIGKV (PAR-2 agonist with 100 μM scrambled peptide VKGILS as control) and MDA-MB-231-derived EV (pretreated with FVIIa and FX). To inhibit PAR-2, endothelial cells were pretreated with 50 μM FSLLRY for 30 min before addition of EV. E-selectin expression **(C)** and IL-8 secretion **(D)** were measured after the treatments (*n* = 3). *p < 0.05, **p < 0.01, ***p < 0.001.

Since FXa has been shown to activate both PAR-1 and PAR-2 (19–21), we sought to determine which of these PARs was involved in the observed endothelial responses, using an agonist and inhibitor approach. We observed an increase in E-selectin expression and IL-8 secretion in E4+ HUVEC with the PAR-1 agonist peptide, TFLLR (Figures 5A,B) but not the scrambled peptide, RLLFT. The canonical PAR-1 agonist, thrombin, also stimulated these endothelial responses (Figure S3C in Supplementary Material). Pretreatment of endothelial cells with a PAR-1 antagonist, E5555, abolished the responses to both the PAR-1 agonist peptide and MDA-MB-231-derived EV (Figures 5A,B). By contrast, a PAR-2 agonist (SLIGKV) or scrambled peptide (VKGILS) had no effect on the endothelial cells; the PAR-2 antagonist (FSLLRY) did not reduce endothelial responses to MDA-MB-231-derived EV (Figures 5C,D). We attribute the lack of PAR-2-mediated response to the weak PAR-2 expression in a subset of the non-activated endothelial cells (Figure 2). To verify that the PAR-2 agonist was functional, we treated MDA-MB-231 (which strongly expresses PAR-1 and PAR-2 as shown in Figure 2) and E4+ HUVEC with the PAR-1 and PAR-2 agonist for 30 min, and evaluated downstream PAR-mediated signaling by immunoblotting for phosphorylated ERK (34) (Figure S4 in Supplementary Material). Phosphorylation of ERK was observed in MDA-MB-231 tumor cells with both PAR-1 and PAR-2 agonists. However, in E4+ HUVEC, ERK phosphorylation was observed with the PAR-1, but not the PAR-2, agonist. Taken together, our data support that the tumor-derived EVs are stimulating the endothelial cells primarily *via* PAR-1. Neither tumor-derived EV nor inhibitors affected endothelial viability (Figure S5 in Supplementary Material), confirming that the endothelial responses were not a consequence of cell death.

## Discussion

In this study, we demonstrate that TF-expressing EVs induce a pro-adhesive and pro-inflammatory phenotype in quiescent endothelial cells. This effect was mediated by TF-dependent generation of FXa and PAR-1 activation. Our results suggest that PAR-1 is the dominant receptor on unstimulated endothelial cells for the TF–FVIIa–FX complex on tumor-derived EV. Endothelial responses induced by FXa have largely been ascribed to PAR-2 (21, 22, 26, 27); however, this was not the case in our study, likely due to the weak PAR-2 expression on the endothelial cells. Our study focused on the 6-h acute response of unstimulated ECs upon exposure to tumor-derived EV. PAR-1 can trans-activate PAR-2 (35), and PAR-2 expression can be upregulated in endothelial cells after several hours of exposure to FXa, inflammatory cytokines, or hypoxic conditions (22). Thus, it is possible that after the initial stimulation by PAR-1, PAR-2 may also play a role in endothelial responses to TF-expressing tumor-derived EV.

Using immunofluorescent microscopy, we observed that the upregulation of E-selectin expression occurred in a distinct subset of cells (~15–40% dependent on agonist). These data suggest that there is heterogeneity within a given endothelial population and that tumor-derived EV may only activate “receptive” endothelial cells by virtue of surface receptor expression or triggering of permissive downstream signaling responses. The mechanisms by which tumor-derived EV bind to or fuse with endothelial cells are not fully elucidated.

The conversion of quiescent endothelial cells to a pro-adhesive and pro-inflammatory phenotype by tumor-derived EV could impact metastasis. Dysfunctional, but not normal, endothelial cells promote tumor inflammation, invasion, and metastasis (36, 37). Endothelial activation could potentially be one of the first steps in which pre-metastatic niches are formed. Upregulation of endothelial adhesion molecules, such as E-selectin, could promote rolling and arrest of bone marrow-derived cells and tumor cells for metastasis (38–40). Increased secretion of IL-8 could induce inflammation, further benefiting metastasis by recruiting pro-inflammatory cells and promoting growth factor and chemokine secretion (41). We investigated E-selectin expression and IL-8 secretion in endothelial cells in response to tumor-derived EV, but other proteins and chemokines that may impact metastasis, such as intracellular adhesion molecule-1 (42) and monocyte chemoattractant protein-1 (43), may be upregulated by tumor-derived EV as well.

Large quantities of high TF-expressing tumor-derived EV in the circulation are likely to generate thrombin, which will activate PAR-1 more strongly than FXa and override any observed FXa-mediated responses (20). Thrombin generation may also trigger paraneoplastic thrombosis (albeit, controversial) (5) or promote thrombosis through impairing anti-tumor immune defenses (44). However, it is more likely that endothelial cells at sites of metastasis are exposed to low concentrations of continuously shed EV over a long period of time versus single large boluses. We speculate that low concentrations of TF-expressing EV may carry a “coat” of FXa, which does not generate thrombin systemically; with continuous exposure and binding of EV to endothelial cells, a threshold concentration required to activate endothelial cells *via* PAR-1 cleavage may be reached. Initial PAR-1-mediated signaling could then “prime” quiescent endothelial cells into a pro-inflammatory phenotype, facilitating pre-metastatic niche formation and metastasis of the primary tumor. Our data suggest that FXa inhibitors (for example, rivaroxaban) could be useful as preventative adjunct therapy in cancer patients to not only reduce cancer-associated thrombosis but also minimize tumor-induced EV endothelial activation (45), which may help reduce metastasis.

## Author Contributions

SC contributed to experimental design, acquisition and analysis of data, and draft of manuscript. JP contributed to acquisition and analysis of data and revision of content. TS contributed to experimental design and draft of manuscript.

## Conflict of Interest Statement

The authors declare that the research was conducted in the absence of any commercial or financial relationships that could be construed as a potential conflict of interest.
