# Supplementary material for: Tissue Factor-Expressing Tumor-Derived Extracellular Vesicles Activate Quiescent Endothelial Cells via Protease-Activated Receptor-1
Source: Front Oncol. 2017 Nov 2;7:261. doi: 10.3389/fonc.2017.00261 (PMC5673848; doi:10.3389/fonc.2017.00261)
Supplement: Supplementary file 1 [file Presentation_1.PDF]

Supplementary Material

Tissue factor-expressing tumor-derived extracellular vesicles activate quiescent endothelial cells via PAR-1

Sara P.Y. Che[1], Jeannie Y. Park[2], Tracy Stokol[2*]

[1]Meinig School of Biomedical Engineering, Cornell University, Ithaca, NY, USA
[2]Department of Population Medicine and Diagnostic Sciences, College of Veterinary Medicine, Cornell University, Ithaca, NY, USA

*** Correspondence:**
Tracy Stokol
tracy.stokol@cornell.edu

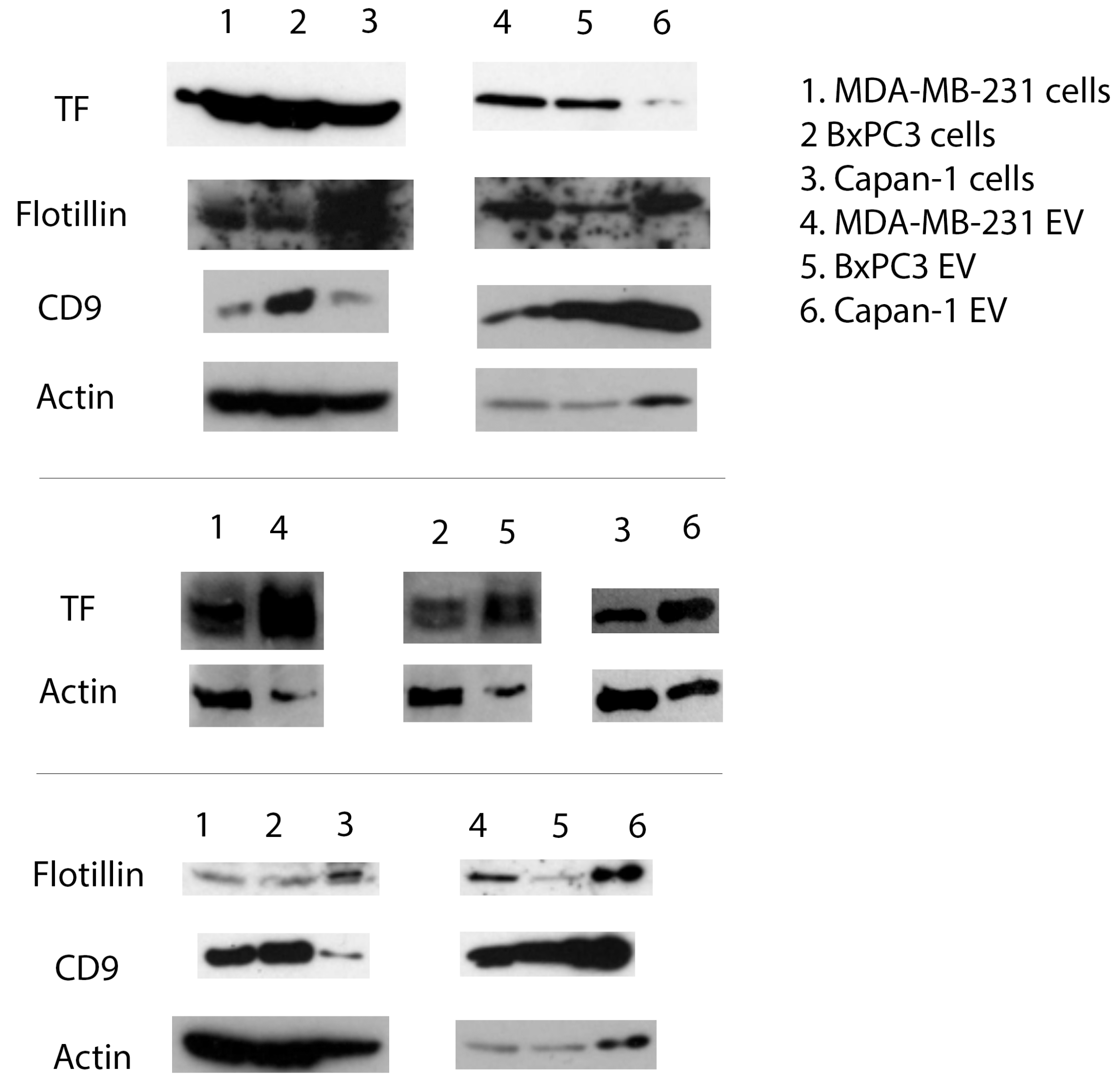

Supplementary Figure 1 Immunoblots of extracellular vesicle (V) markers on whole cell lysates of tumor cell lines and derived EV. Numbers 1-3 and 4-6 correspond to whole cell lysates and EV of the designated cell lines, respectively.

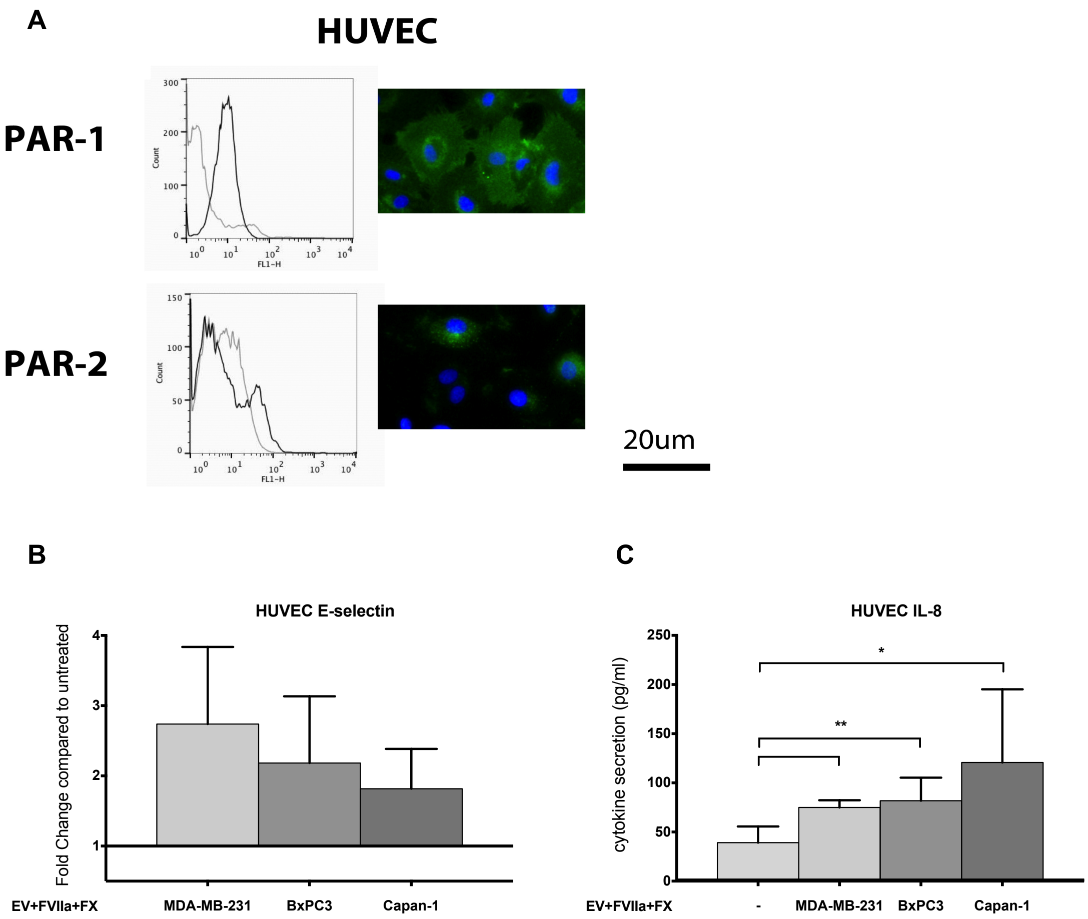

Supplementary Figure 2 Characterization of PAR expression on non-activated HUVEC and E-selectin expression and IL-8 secretion after exposure to tumor-derived EV (pretreated with FVIIa and FX). A. Expression of PAR-1 and PAR-2 on HUVEC was evaluated using flow cytometry (black line: protein of interest; grey line: isotype control) and immunofluorescent microscopy (green: protein of interest; blue: nuclear counterstain). Images are representative of three separate experiments. **B and C.** HUVEC were exposed to EV (with FVIIa and FX) derived from MDA-MB-231, BxPC3 and Capan-1 tumor cells for 6 hours, then endothelial E-selectin expression (B) and IL-8 secretion (C) were evaluated using a cell-based ELISA and ELISA on conditioned media, respectively (n=3).

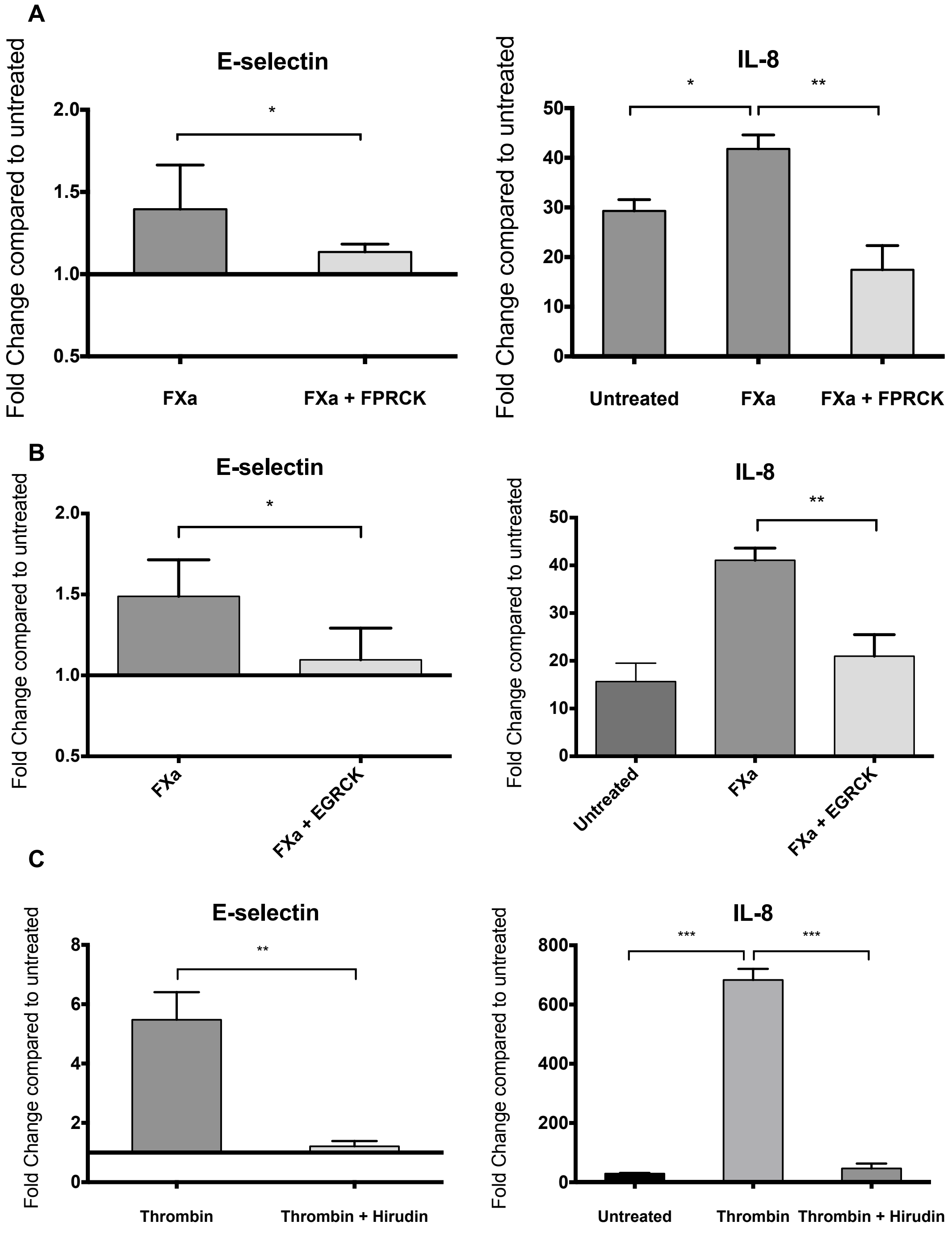

Supplementary Figure 3. Endothelial responses to purified FXa and thrombin. E4+ HUVEC were treated with 80nM FXa, which weakly induced endothelial E-selectin expression and IL-8 secretion. The responses were abolished by **A.** 40μM FPRCK and **B.** 40μM EGRCK. **C.** E-selectin expression and IL-8 secretion by E4+ HUVEC was stimulated by 1U/ml thrombin, and the responses were abolished with 10U/ml hirudin (n=3). * $p<0.05$, ** $p<0.01$, *** $p<0.001$.

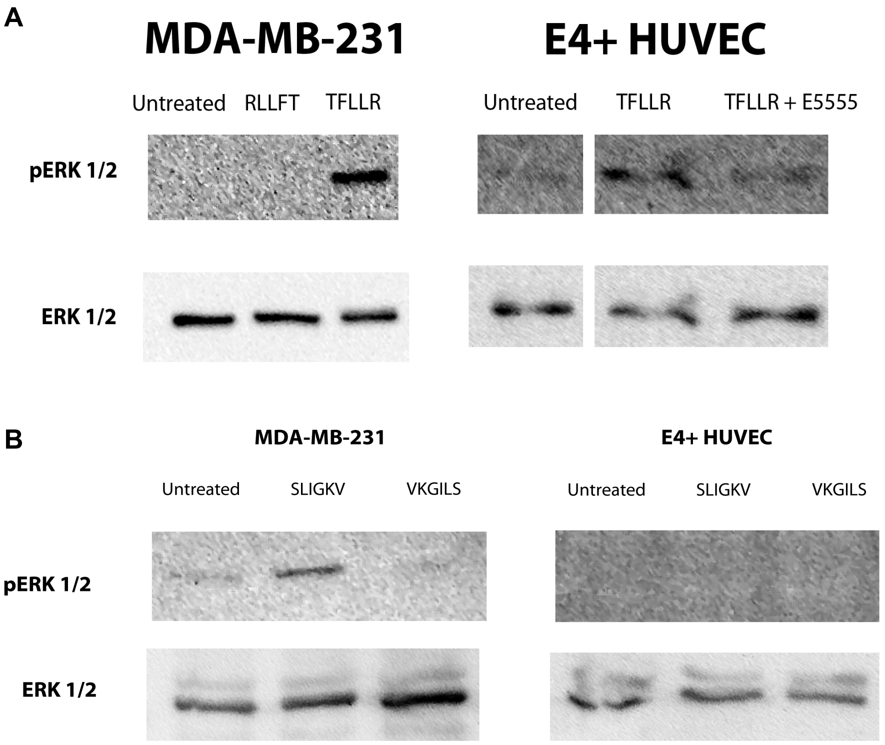

Supplementary Figure 4. Phosphorylation of ERK in MDA-MB-231 and E4+ HUVEC lysates with PAR-1 and PAR-2 agonists. E4+ HUVEC and MDA-MB-231 (positive control for PAR-1 and PAR-2) were treated with **A.** PAR-1 and **B.** PAR-2 agonist (100μM TFLLR or SLIGKV, respectively) with scrambled agonists (100μM RLLFT or VKGILS, respectively) as negative controls. Total ERK1/2 was used as a loading control. **A**. Immunoblots of lysed cells showed phosphorylation of ERK1/2 with PAR-1 agonists in E4+HUVEC and MDA-MB-231. The ERK phosphorylation in E4+HUVEC was inhibited by pretreatment with the PAR-1 antagonist, E-5555 (0.1μM) for 30 minutes. **B.** ERK phosphorylation was only seen in MDA-MB-231 with the PAR-2 agonist peptide. Images are representative of 3 independent experiments.

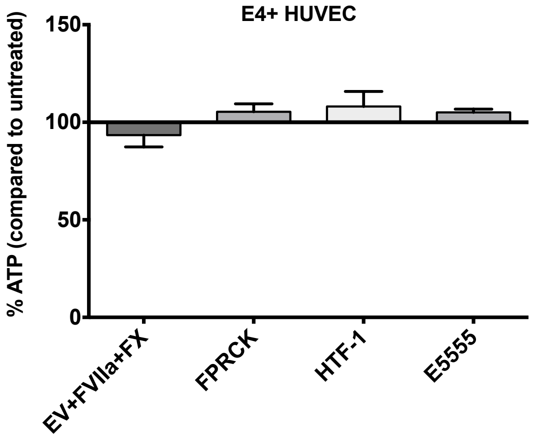

Supplementary Figure 5. MDA-MB-231-derived EV (pretreated with FVIIa and FX) and chemical or immunologic inhibitors of FXa (FPRCK, 40μM), TF (antibody HTF-1, 10μg/mL), or PAR-1 (E5555, 0.1μM) does not induce endothelial cell death. Cell viability was evaluated after treating E4+ HUVEC exposed to MDA-MB-231-derived EV or the various inhibitors for 6 hours. Data is presented as percentage compared to untreated cells (n=3).
